# Cephalosporin resistance in community acquired spontaneous bacterial peritonitis

**DOI:** 10.12669/pjms.35.1.17

**Published:** 2019

**Authors:** Shahid Sarwar, Shandana Tarique, Umaima Waris, Anwaar A. Khan

**Affiliations:** 1 Shahid Sarwar MBBS, FCPS (Medicine), (Gastroenterology) MCPS-HPE. Associate Professor, Services Institute of Medical Sciences, Consultant Gastroenterologist, Doctors Hospital & Medical Center, Doctors Hospital & Medical Center Lahore, Pakistan; 2 Shandana Tarique, MBBS, FCPS (Medicine). Professor of Medicine, King Edward Medical University, Lahore, Pakistan. Doctors Hospital & Medical Center Lahore, Pakistan; 3 Dr. Umaima Waris Post-Graduate Resident Medicine, Services Institute of Medical Sciences, Doctors Hospital & Medical Center Lahore, Pakistan; 4 Anwaar A. Khan, MACP, FACG, FRCP, FCPS. Consultant Gastroenterologist, Doctors Hospital & Medical Center Ex- Dean and Professor of Gastroenterology, Shaikh Zayed Post Graduate Medical Institute, Lahore, Pakistan

**Keywords:** Cephalosporins, In-hospital mortality, Resistance, Spontaneous bacterial peritonitis

## Abstract

**Objective:**

To determine 3^rd^ generation cephalosporin resistance in patients with community-acquired spontaneous bacterial peritonitis (SBP) using early response assessment.

**Methods:**

This prospective quasi-experimental study was carried out at Doctors Hospital & Medical Center from January 2016 to September 2018. Patients with cirrhosis and SBP were included. Third generation cephalosporins i.e. cefotaxime/ceftriaxone were used for treatment of SBP. Response after 48 hours was assessed and decline in ascitic fluid neutrophil count of < 25% of baseline was labelled as cephalosporin resistant. Carbapenem were used as second line treatment. Recovery and discharge or death of patients were primary end points.

**Results:**

Male to female ratio in 31 patients of SBP was 1.2/1 (17/14). Hepato-renal syndrome was diagnosed in 11(37.9%) patients. Cefotaxime was used for 16(51.6%) patients whereas ceftriaxone for 15(48.3%) patients. Early response of SBP was noted in 26(83.8%) patients while 5 (16.2%) were non-responders to cephalosporins. SBP resolved in all non-responding patients with i/v carbapenem. In-hospital mortality was 12.9% and had no association with cephalosporin resistance. High bilirubin (p 0.04), deranged INR (p 0.008), low albumin (p 0.04), high Child Pugh (CTP) score (p 0.03) and MELD scores (p 0.009) were associated with in-hospital mortality.

**Conclusion:**

Cephalosporin resistance was present in 16.2% of study patients with community-acquired SBP. Mortality in SBP patients is associated with advanced stage of liver disease.

## INTRODUCTION

Spontaneous bacterial peritonitis (SBP) is a dreaded complication in patients with decompensated cirrhosis. A prospective study concluded that 47% patients with cirrhosis being admitted in hospital have bacterial infections and 31% of them have SBP.^1^ It is considered a lethal infection with one month mortality of 32% and one year mortality of 60% despite optimum treatment.^2^ It is the outcome of increased intestinal bacterial growth, easy translocation across intestine along with impaired host immune response in a patient with cirrhosis of liver.^3^

Every patient being admitted with ascites should undergo diagnostic paracentesis to diagnose SBP. Mortality rate of patients who underwent paracentesis at admission was lower than the patients who did not have ascitic fluid analysis (6.3% vs 8.9%) highlighting the possibility of missing the diagnosis of SBP.^4^ Another study concluded that each hour delay in paracentesis of admitted patients of cirrhosis related ascites, increases mortality by 3.3%.^5^ Diagnosis of SBP is based on absolute neutrophil count in ascitic fluid and count above 250/mm^3^ is considered diagnostic for SBP. Cultures are positive in only 40% of patients which can be increased to 80% with bedside inoculation of fluid in culture bottle.^6^

Due to low yield of fluid culture and high mortality, early empirical treatment with antibiotics is recommended. Karvellas et al, noted that every hour delay in starting antibiotics increases mortality by 1.86 times.^7^ Choice of antibiotic is dependent on type of microbes responsible for infection. Gram negative enteric bacteria are considered the most common pathogens responsible for SBP. This is the reason, 3^rd^ generation cephalosporins are the recommended drugs of choice for treating SBP empirically.^8^ But recent studies have shown that Cephalosporins are effective only in 70% of community acquired and 56% of hospital acquired SBP.^6^ It is most likely due to changing bacterial pathogens of SBP over last two decades as now gram positive bacteria and multi drug resistance organism (MDRO) are increasingly being isolated in SBP.^9^ It is the consequence of undue, over the counter misuse of cephalosporins in community and frequent exposure of cirrhosis patients to these drugs during recurrent hospital admissions.

This emerging trend of resistance, is the reason, many international guidelines are recommending selection of drugs in light of regional prevalence of micro-organisms responsible for SBP and antibiotic resistance patterns.^10^ However, due to low ascitic fluid culture yield, treatment may be guided by early decline in PMN count after starting antibiotic therapy. Recent EASL guidelines have also recommended use of early response assessment i.e. decline of neutrophil count in ascitic fluid of ≥ 25% of baseline count after 48 hours of antibiotics therapy for guidance, regarding treatment response.^10^

There is paucity of data on the prevalence of micro-organisms responsible for SBP in patients of cirrhosis in our region, there is no community based data on antibiotics resistance to guide our management decisions. Unwarranted use of antibiotics is much more prevalent in our society as compared to western world.^11^ Therefore, we are more likely to find MDROs in our patients with SBP. We need to determine drug resistance patterns, especially for cephalosporins which are still first line recommended drugs for SBP. Due to very low yield of ascitic fluid culture, early response assessment in SBP patient can be a good tool to guide our treatment regimen.

We planned a study to determine 3^rd^ generation cephalosporin resistance in our patients with spontaneous bacterial peritonitis using early response assessment to guide treatment outcome.

## METHODS

This prospective quasi-experimental study was carried out at Doctors Hospital & Medical Center from January 2016 to September 2018. Patients of liver cirrhosis being admitted with ascites were provisionally included in the study. Cirrhosis of liver was diagnosed on the basis of coarse and nodular texture of liver on ultrasound examination. Patients who refused ascitic fluid paracentesis, those with ascites secondary to causes other than cirrhosis like tuberculosis, malignancy, congestive cardiac failure, kidney disease etc were excluded. Patients who developed SBP more than 48 hours after admission as confirmed by negative initial ascitic fluid report were also excluded.

Ascites was graded as mild, only detectable on ultrasound examination, moderate, with moderate symmetrical abdominal distension and tense, if abdomen is grossly distended.^10^ Paracentesis of ascitic fluid was carried out under ultrasound guidance by drawing at least 50 ml of fluid using standard aseptic techniques for differential count, biochemistry, cytology and culture. Diagnosis of SBP was confirmed if absolute neutrophil count in ascitic fluid was more than 250/mm.^3^ Only patients confirmed to have SBP on ascitic fluid analysis at admission were included.

Laboratory investigations including complete blood count, liver function tests, coagulation profile, serum electrolytes, renal function tests were done on admission. Child Pugh Turcotte (CTP) and Model for End stage Liver Disease (MELD) were used for staging of liver disease. Sequential Organ Failure Assessment (SOFA) score was used to assess severity of infection.

All patients were randomly grouped using online random table generator Stat Trek^®^ as A and B. Group A patients received intravenous 3^rd^ generation cephalosporin, cefotaxime eight grams/day in 4 divided doses whereas group B patients were treated with ceftriaxone two gram/day. Intravenous albumin 20 grams/day was given to all patients for five days. Patients with hepato-renal syndrome Type-1 were also given Inj Terlipressin 4mg/day with gradual dose escalation, if no response, up to maximum of 12 mg/day. Electrolyte disturbances, variceal bleeding or hepatic encephalopathy if present were managed as per standard protocol.

Patients were daily monitored for symptoms, vital signs, fluid intake/output, serum electrolytes, renal functions and progress of hepatic encephalopathy or GI bleed if present. Repeat paracentesis was performed after 48 hours of treatment and fluid was checked for absolute neutrophil count. Reduction in neutrophil count ≥ of 25% of base line count was considered as positive early response of SBP treatment, same treatment was continued for five days. Patients in whom Absolute neutrophil count failed to decline by 25% or more were labeled as Cephalosporin resistant and their treatment was changed based on ascitic fluid culture and sensitivity report.

In patients with negative ascitic fluid culture report, I/V carbapenem group of drugs with dose modification for creatinine clearance <50ml/min were given. Follow-up paracentesis was done again in all patients with treatment change to verify response to therapy. Primary outcome measure was presence or absence of early response to cephalosporins whereas discharge from hospital or death during hospital admission were secondary outcome measures.

### Statistical Analysis

Data were analyzed using SPSS^®^ 20. Numerical variables like age, CTP score, MELD score etc were expressed as mean ± standard deviation (SD) and categorical variables like grades of ascites etc were given in percentage for normally distributed variables whereas median and interquartile range (IQR) for variables not normally distributed. Shapiro-wilk test was used for checking whether variables were normally distributed or not.

Unpaired student’s t test was used to compare numerical variables while χ^2^ test was used to compare categorical variables. Mann-Whitney U test was used for variables not normally distributed. P value of ≤0.05 was considered statistically significant.

## RESULTS

Thirty-one patients with confirmed diagnosis of spontaneous bacterial peritonitis were included. Male to female ratio was 1.2:1(17/14). Fever was presenting complaint in 19(61.3%) patients, 28(90.3%) patient had worsening ascites, 13(41.9%) had hepatic encephalopathy at time of admission and 3(9.7%) had variceal bleeding. Ascites was mild in 2(6.5%) patients, moderate in 21(67.7%) and tense in 8(25.8%) patients. Pleural effusion was present in 6(19.4%) patients. Hepato-renal syndrome (HRS) was diagnosed in 11(35.5%) patients, 5(16.1%) had HRS Type-1 and 6(19.4%) had HRS type 2.

Liver cirrhosis was CTP class B in 10(32.3%) patients whereas remaining 21(67.7%) were in CTP class C. MELD score was 20 or more in 20(64.5%) patients whereas 3(9.6%) patients had SOFA score above nine. Hepatocellular carcinoma was present in 7 (22.5%) patients. Ascitic fluid culture was positive only in one patient for E. Coli.

Cefotaxime treatment was given in 16(51.6%) patients of group A whereas, 15(48.3%) patients in group B were treated with ceftriaxone. All patients received I/V albumin and 5(16.1%) received terlipressin for HRS Type-1.

Decline of ≥ 25% in absolute neutrophil count in ascitic fluid was achieved in 26(83.8%) patients within 48 hours while 5(16.2%) patients failed to achieve early response to treatment, 2 (12.5%)in group A and 3 (20%) in group B and difference was insignificant (p value 0.57) and were labelled as Cephalosporin resistant patients. Antibiotic was changed to Meropenem in 4(12.9%) patients whereas, imipenem and cilastatin sodium (Tienam) in one patient for treating cephalosporin resistant SBP. All 5 patients had resolution of infection with new antibiotics as confirmed on repeat paracentesis.

Of five patients with HRS Type-1, 4(80%) responded to treatment while one patient died despite hemodialysis. Of patients included, 4(12.9%) died during hospital admission, 2(6.4%) due to worsening encephalopathy, one (3.4%) following HRS and 1(3.2%) as a result of acute on chronic liver failure and none due to non-resolution of SBP. Mortality was noted in 1 (6.2%) patient of group A and in three (20%) group B patient and again difference was insignificant (p value 0.25)

We compared patients with and without cephalosporin resistance as shown in Table-I. Patients in both group were similar in terms of severity of liver disease suggesting that resistance to medication is not an outcome of stage of liver disease but most likely is consequence of type of organisms responsible for SBP.

**Table-I:** Comparison of patients with and without cephalosporin resistance.

Variables	Cephalosporin sensitive patients(n-24) Mean values (±SD)	Cephalosporin resistant patients (n- 5) Mean values (±SD)	P value
Age (years)	56.2(10.6)	52.4 (9.6)	0.45
TLC (x 10^3^/µL)	11.2 (4.9)	13.9 (4.2)	0.24
Platelet (x 10^9^/L)	140.5 (94.8)	145.6 (61.7)	0.91*
PT (sec)	21.4 (8.6)	18.6 (3.7)	0.49
Bilirubin (mg/dl)	4.6 (4.92)	6.7 (8.6)	0.8 *
Albumin (g/dl)	2.4 (0.46)	2.2 (0.16)	0.36
Creatinine (mg/dl)	1.52 (0.9)	2.26 (2.14)	0.20
CTP score	10.7 (2.05)	11.2 (1.9)	0.61
MELD score	23.4 (8.3)	24.8 (9.5))	0.73
SOFA score	4.85 (3.3)	6.6 (3.9)	0.30

* Mann Whitney U test

We also had comparative analysis of patients who died and those who recovered of acute illness as shown in Table-II. In-hospital death in patients with SBP was significantly associated with deranged INR (p value 0.008), raised bilirubin (p value 0.04), low albumin (p value 0.04), high CTP score (p value 0.03), worse MELD score (p value 0.009) and SOFA score > 9 (p value <0.00). Mortality in patients with SBP was related to severity of underlying liver cirrhosis but was not a consequence of cephalosporin resistance.

**Table-II:** Patient’s variables associated with in-hospital mortality.

Variables	Patients who died (n-4) (Mean± SD)	Patients who recovered (n-25) (Mean± SD)	P value
Age	53.5 (14.8)	55.9 (10.0)	0.66
TLC (x 10^3^/µL)	15.8 (2.7)	11.0 (4.7)	0.06
Platelet (x 10^9^/L	109.5 (73.7)	146.1 (91.7)	0.45
INR	2.68 (1.3)	1.59 (0.58)	0.008
Bilirubin (mg/dl)	10.3(8.6)	4.13 (4.6)	0.04*
Albumin (g/dl)	1.97 (0.09)	2.44 (0.43)	0.04
Creatinine (mg/dl)	2.7 (2.3)	1.48 (0.88)	0.04
CTP score	12.75 (0.95)	10.5 (1.9)	0.03
MELD score	33.5 (2.3)	22.1 (7.9)	0.009
SOFA score	11.25 (2.9)	4.22 (2.4)	0.00

* Mann Whitney U test

## DISCUSSION

Antibiotic resistance is an emerging issue being faced in every field of medicine. It is the outcome of wide spread use of antibiotics over the past few decades. Irrational use of antibiotics and self-medication are major contributors in rise of drug resistance. Now we have terminologies like multi-drug resistance (MDR), extensive drug resistance (XDR)^12^ and Super Bugs, each highlighting the magnitude of problem.

Management of potentially lethal complication of SBP in patients at advanced stage of liver disease is being adversely affected by increasing drug resistance. In this study more than 16% of patients with community acquired SBP were resistant to first line treatment, 3^rd^ generation cephalosporins with no difference in efficacy of cefotaxime and ceftriaxone. In a study of 192 cases of SBP, overall prevalence of cephalosporin resistance was 19%, 8% for community acquired and 41% for nosocomial acquisition.^13^ Alexopoulou A, et al reviewed 47 patients with SBP, 60% of them were healthcare-associated infections. Cephalosporin resistance was 49% and quinolone resistance was 47% while multi-drug resistance was seen in 19% of isolated bacteria.^14^

Increasing resistance to cephalosporins is most likely due to changing microbiological flora responsible for SBP. Gram negative bacteria were traditionally regarded as predominant etiology for SBP, however it is now being questioned. Gram positive cocci were responsible for 45.9% of 140 cases of SBP followed by enterobacteriaceae in 31.7% cases in a study by Novovic S et al.^15^ Cephalosporin coverage for SBP flora was only 57% in this study. In a 10 years interval study by Oey RC et al, prevalence of gram positive bacteria in culture positive SBP increased from 26% to 46%.^16^ Only one patient of SBP was culture positive in our study. Majority of these patients are already on antibiotics either prescribed by general physicians or due to self-medication resulting in negative culture results. Only 25% patients of SBP were culture positive in another local study by Sajjad M et al.^17^

We have not found any association between stage of liver disease and cephalosporin resistance in our study. Ariza X, et al identified previous use of cephalosporins, diabetes mellitus, upper GI bleeding and nosocomial acquisition as risk factors for resistance but no association of degree of liver dysfunction with drug resistance. They noted increased mortality in patients with cephalosporin resistance.^18^ In a Korean study of 188 community acquired SBP patients, risk factors for 30 days all-cause mortality showed high CTP score, acute kidney injury and resistance to 3^rd^ generation cephalosporins.^19^ Presence of hepatic encephalopathy and hepatocellular carcinoma were independent predictors for 30 days mortality in SBP patients in a study of 1176 SBP patients from Taiwan.^20^ Association of mortality and antibiotic resistance is mostly seen in nosocomial infections and less so in community acquired SBP.^21^ Indicators of worsening liver disease like high bilirubin, low albumin, deranged INR, high CTP and MELD scores were associated with in-hospital mortality in our study but no association of cephalosporin resistance was noted with mortality.

In this new paradigm, of changing SBP flora and emergence of drug resistance, combination of antibiotics instead of single agent are being advocated for treating SBP.^22^ European Association for Study of Liver has recommended in its guidelines of 2018 to consider disease severity and regional resistance pattern while choosing antibiotics. Piperacillin/Tazobactam is considered first line drug for both community acquired and nosocomial infections even for areas with low prevalence of MRDO. Cephalosporin are still an option for community acquired but no more for health care associated or nosocomial infection. Two or more drugs combination is recommended for areas with high prevalence of drug resistance.^10^

Since we are increasingly encountering resistance to treatment in SBP, we therefore, need to modify treatment strategies. We need further data on prevalent microbial flora, resistance patterns and efficacy of alternative drugs available. Our study results will serve to guide such decisions in future.

Our data is limited by predominantly negative culture results of ascitic fluid, a real life situation where mostly choice of drugs is empirical. We have used early response assessment at 48 hours of treatment to guide timely treatment modification. It is an effective tool for guiding treatment decisions especially in patients of SBP with negative culture results. Its use should be encouraged in settings with limited resources for expensive culture studies and poor standardization of these tests in the presence of high risk for resistance.

### Conclusions

Third generation Cephalosporin resistance is present in 16.2% of patients with community acquired spontaneous bacterial peritonitis. Mortality in SBP patients is associated with worsening stage of liver disease but not with cephalosporin resistance.

### Authors’ Contribution

**SS:** Conceived, designed, did statistical analysis and manuscript writing.

**SS, ST,** **&** **UW:** Did data collection and manuscript review.

**AAK:** Did manuscript review and final approval.
